# Disseminated Bartonella henselae Infection in a Patient With Relapsed/Refractory Multiple Myeloma Following Autologous Stem Cell Transplantation

**DOI:** 10.7759/cureus.95516

**Published:** 2025-10-27

**Authors:** Sanjay Muttineni, Sasya Dronavalli, Sharmilan Thanendrarajan, Frits Van Rhee, Juan Carlos Rico Crescencio, Nikhil Verma

**Affiliations:** 1 Internal Medicine, University of Arkansas for Medical Sciences, Little Rock, USA; 2 Hematology and Medical Oncology, University of Arkansas for Medical Sciences, Little Rock, USA; 3 Infectious Disease, University of Arkansas for Medical Sciences, Little Rock, USA

**Keywords:** autologous stem cell transplant, bartonella, cat-scratch disease, fever of unknown origin, immunocompromised patient, relapsed and refractory multiple myeloma

## Abstract

*Bartonella henselae* is the causative agent of cat scratch disease (CSD), a zoonotic illness primarily transmitted to humans through scratches or bites from cats and, less commonly, by cat fleas or lice. While the most common presentation is self-limited regional lymphadenopathy in immunocompetent individuals, in immunocompromised patients, such as organ transplant recipients, it can cause systemic infections.

This report describes a case of disseminated *B. henselae* infection in a 66-year-old man with relapsed/refractory multiple myeloma following conditioning chemotherapy and autologous stem cell transplantation (ASCT). *B. henselae* infection has not been well characterized in patients after ASCT. The patient presented with persistent fever and involvement of the liver and right inguinal lymph nodes after transplantation. Diagnosis was challenging due to nonspecific symptoms and initially negative routine tests; however, detection of *B. henselae* DNA through polymerase chain reaction (PCR) in liver and lymph node biopsy specimens confirmed the infection. Following initiation of antibiotic therapy, the patient showed both symptomatic and radiological improvement. *Bartonella* infections remain poorly characterized in post-transplant patients. This case highlights the diagnostic challenges of atypical infections in immunocompromised individuals, particularly ASCT recipients, and underscores the importance of specialized diagnostic testing, including tissue PCR, for early diagnosis and targeted antimicrobial therapy.

## Introduction

*Bartonella henselae* is a zoonotic pathogen and the causative agent of cat scratch disease (CSD) [1]. Cats are the major reservoir for *B. henselae* [1]. *Bartonella* species are fastidious, intracellular, gram-negative bacteria that invade hematopoietic progenitor cells but do not affect erythroid differentiation, resulting in intraerythrocytic presence and replication [1,2]. The immune response to *B. henselae* infection depends on the individual's immune status [1].

CSD typically presents as a self-limiting, flu-like illness characterized by an erythematous papule at the site of injury 3-10 days after inoculation, followed by localized lymphadenopathy [1,2]. Immunodeficient patients are at risk for disseminated infection and bacillary angiomatosis, which manifests as cutaneous angiogenic lesions [1,2]. Immunocompromised patients may develop rare complications, such as culture-negative endocarditis, myocarditis, granulomatous hepatitis, and neuroretinitis [1-3]. Abdominal imaging can identify hepatosplenic involvement in *Bartonella* infection [2]. Isolation of *Bartonella* in culture is difficult; thus, serological tests such as the indirect fluorescence assay (IFA) and enzyme immunoassay (EIA) are often used for initial screening and diagnosis [1,2]. Direct visualization and polymerase chain reaction (PCR) can provide confirmation [1-3]. Establishing *Bartonella* infection typically requires a combination of diagnostic approaches, including serological assays, PCR-based methods, immunohistochemistry, and/or histopathology [1-3]. Treatment varies according to clinical manifestations, the patient’s immune status, and the organ systems involved [2]. *Bartonella* infections are rare and not well characterized in autologous stem cell transplant (ASCT) recipients. These infections may present with atypical or nonspecific clinical features, making diagnosis particularly challenging. A high index of suspicion and early recognition are critical, as specialized diagnostic testing is often necessary for accurate identification. Early diagnosis and prompt initiation of appropriate antibiotic therapy in disseminated *B. henselae* infection can reduce morbidity and mortality in post-ASCT patients. To the best of our knowledge, this is the first reported case of systemic *B. henselae* infection in a patient with multiple myeloma following high-dose chemotherapy and ASCT.

## Case presentation

A 66-year-old man with high-risk, relapsed/refractory IgG kappa multiple myeloma and biopsy-confirmed extramedullary disease (EMD) in the thyroid cartilage underwent ASCT in February 2025, following high-dose (HD) conditioning chemotherapy with carmustine (BCNU), etoposide, cytarabine, dexamethasone, and melphalan (BEAM). His initial post-transplant course was complicated by a fever of unknown origin (FUO) and elevated C-reactive protein (CRP), which were managed with empiric antibiotics. The patient improved and was discharged.

In March 2025, on Day +15 post-transplant, he returned with a high-grade fever (104°F), chills, headache, fatigue, nasal drip, mild cough, and diarrhea. Physical examination was unremarkable. Laboratory testing revealed a normal leukocyte count with severe lymphopenia, normal liver function tests, normal lactic acid, and elevated inflammatory markers, including CRP and procalcitonin (Table 1).

**Table 1:** Laboratory results on presentation

Parameter	Units	Patient value	Reference range
White blood cell (WBC)	K/uL	3.87	3.60-9.50
Hemoglobin	g/dL	10.5	13.0-17.0
Neutrophils	%	64.4	35.0-65.0
Lymphocytes	%	5.1	23.0-50.0
C-reactive protein (CRP)	mg/L	40.90	0.00-10.00
Procalcitonin	ng/ml	0.38	0.00-0.10
Sodium	mmol/L	132	135-145
Potassium	mmol/L	3.8	3.5-5.1
Chloride	mmol/L	97	98-107
Bicarbonate	mmol/L	24	22-32
Serum creatinine	mg/dl	1.0	0.6-1.3
Blood urea nitrogen (BUN)	mg/dl	7	6-20
Lactate	mmol/L	1.4	0.5-2.2
Alkaline phosphatase (ALP)	IU/L	61	32-91
Gamma-glutamyl transferase (GGT)	IU/L	26	7-50
Lactate dehydrogenase (LDH)	IU/L	320	100-248
Aspartate aminotransferase (AST)	IU/L	51	15-41
Alanine aminotransferase (ALT)	IU/L	39	4-45

Respiratory viral PCR and blood cultures were negative. Clostridium difficile PCR was positive, and oral vancomycin 125 mg four times daily was initiated.

**Figure 1:**
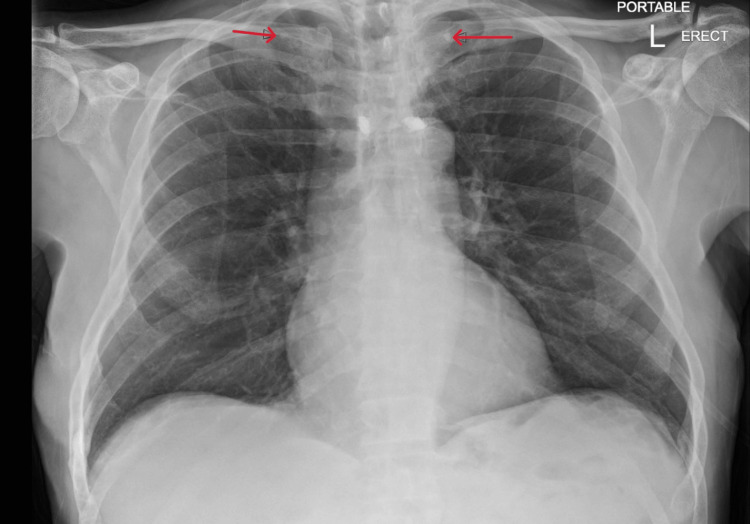
Chest X-ray showing new bilateral apical calcified granulomas

**Figure 2:**
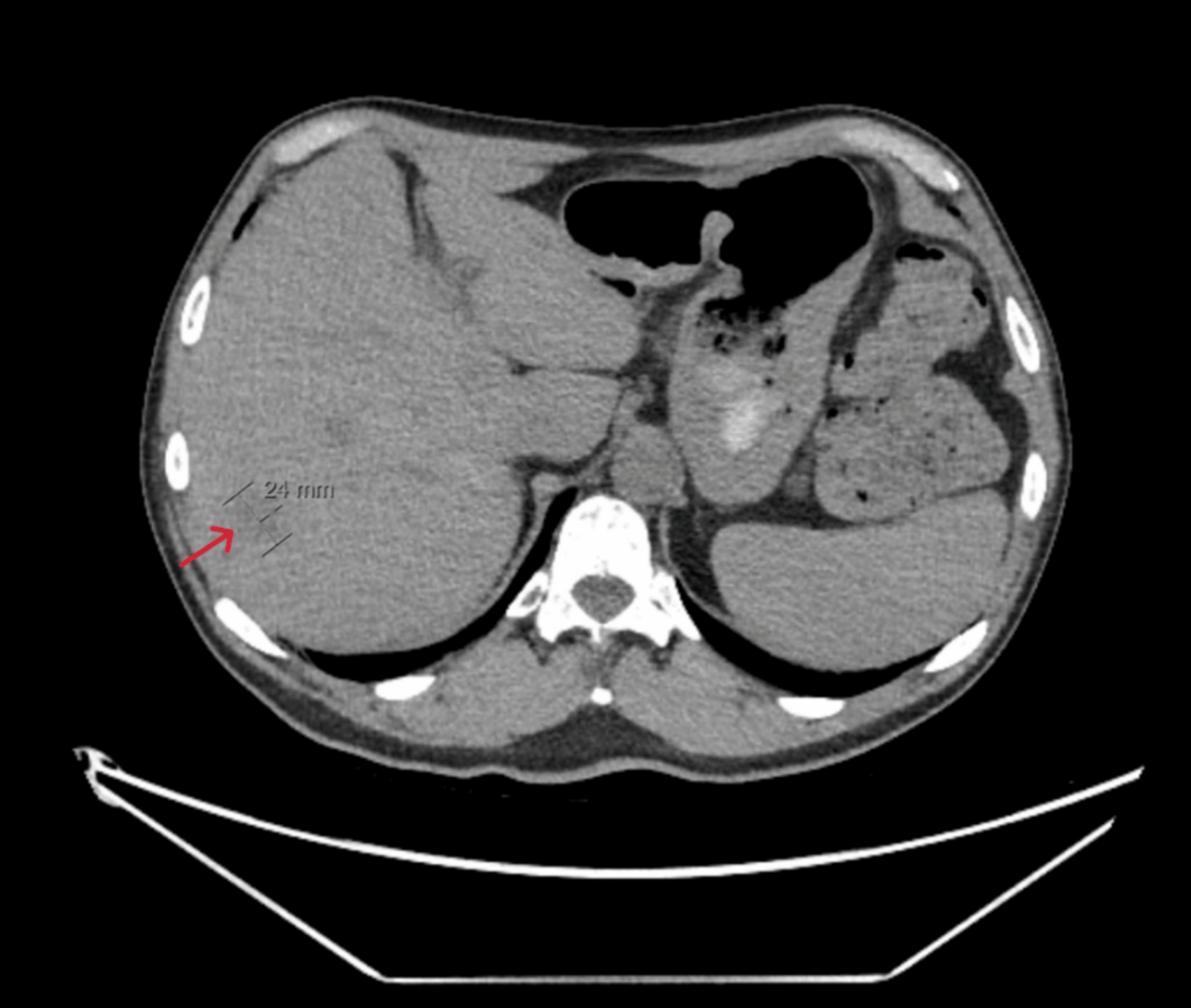
Non-contrast chest CT demonstrating new ill-defined, low-attenuation lesions throughout the liver, measuring up to 2.4 cm in the right hepatic lobe

**Figure 3:**
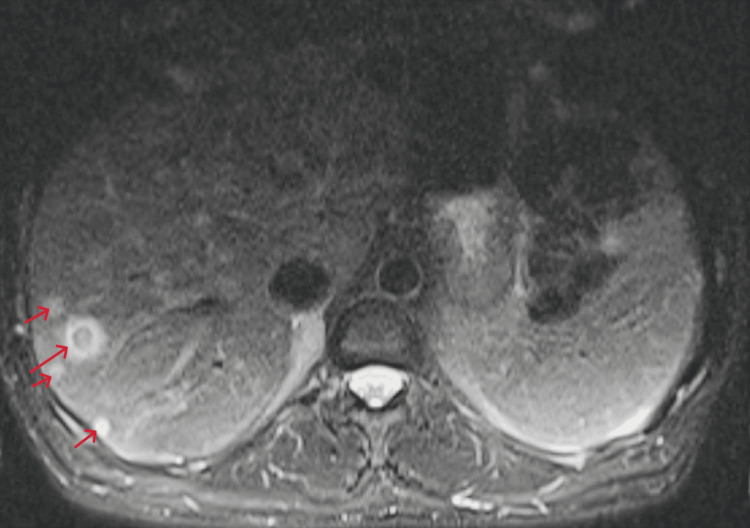
Abdominal MRI with and without contrast showing innumerable T2-hyperintense lesions demonstrating a halo sign, rim enhancement, and central diffusion restriction, concerning for micro-abscesses

**Figure 4:**
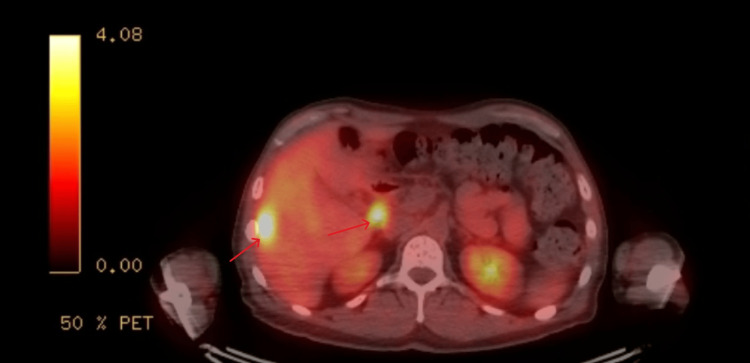
PET-CT showing multifocal, intensely FDG-avid lesions in the right hepatic lobe and an intensely FDG-avid 1.6 cm porta hepatis lymph node

**Figure 5:**
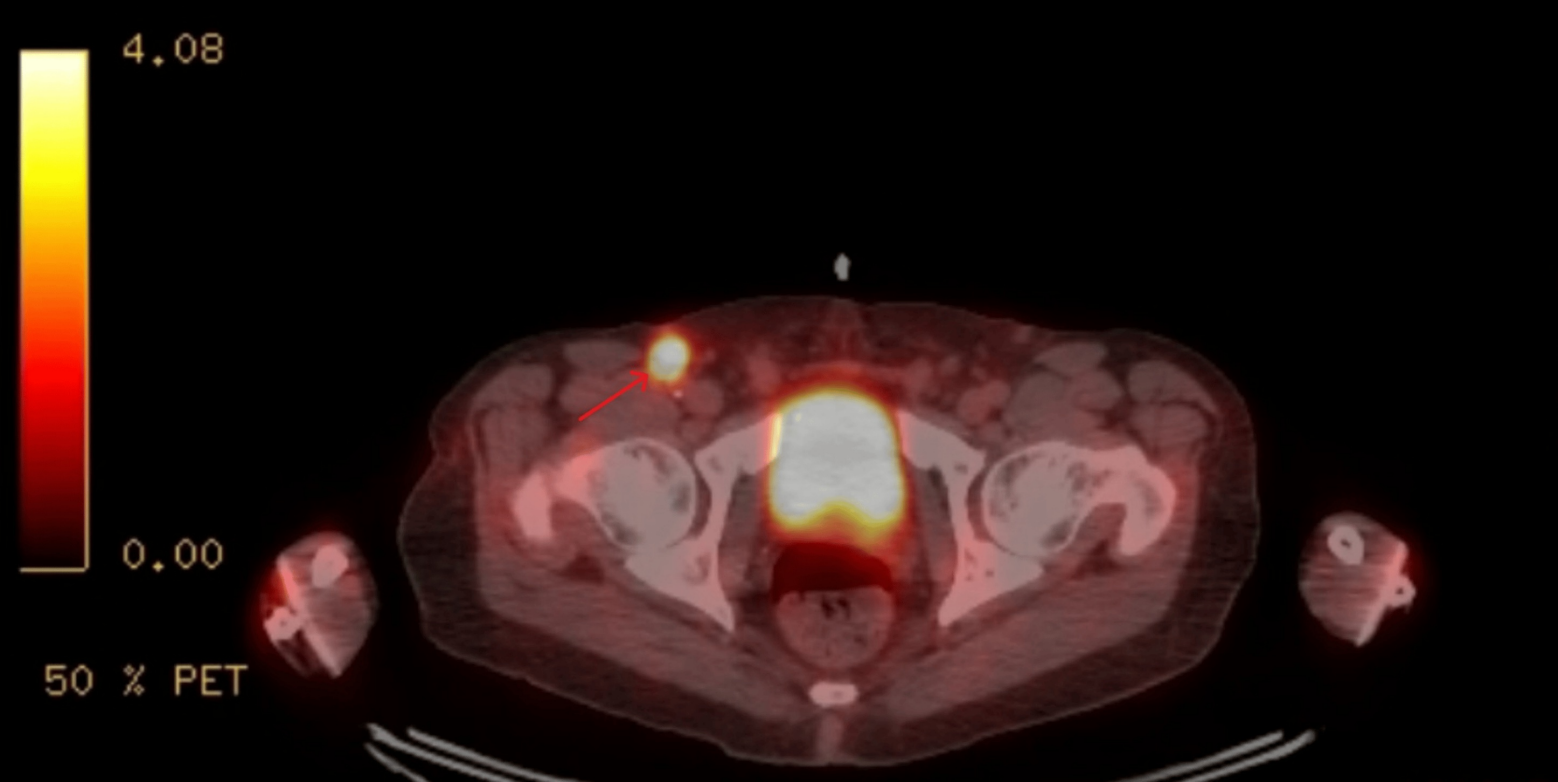
PET-CT showing an intensely FDG-avid 1 cm right inguinal lymph node

Chest X-ray revealed new apical calcified granulomas (Figure 1). Chest CT showed no pulmonary infiltrates but identified new ill-defined hypodense liver lesions (Figure 2). Abdominal MRI demonstrated multiple T2-hyperintense hepatic lesions with rim enhancement and diffusion restriction, concerning for micro-abscesses (Figure 3). PET-CT confirmed FDG-avid liver lesions and lymphadenopathy (Figure 4 and Figure 5).

CT-guided liver biopsy showed necrotizing granulomatous inflammation and micro-abscesses. Acid-fast bacilli (AFB), Gomori methenamine silver (GMS), and cytomegalovirus (CMV) stains were negative, and cultures yielded no growth. Oral amoxicillin-clavulanate was initiated for six weeks.

**Figure 6:**
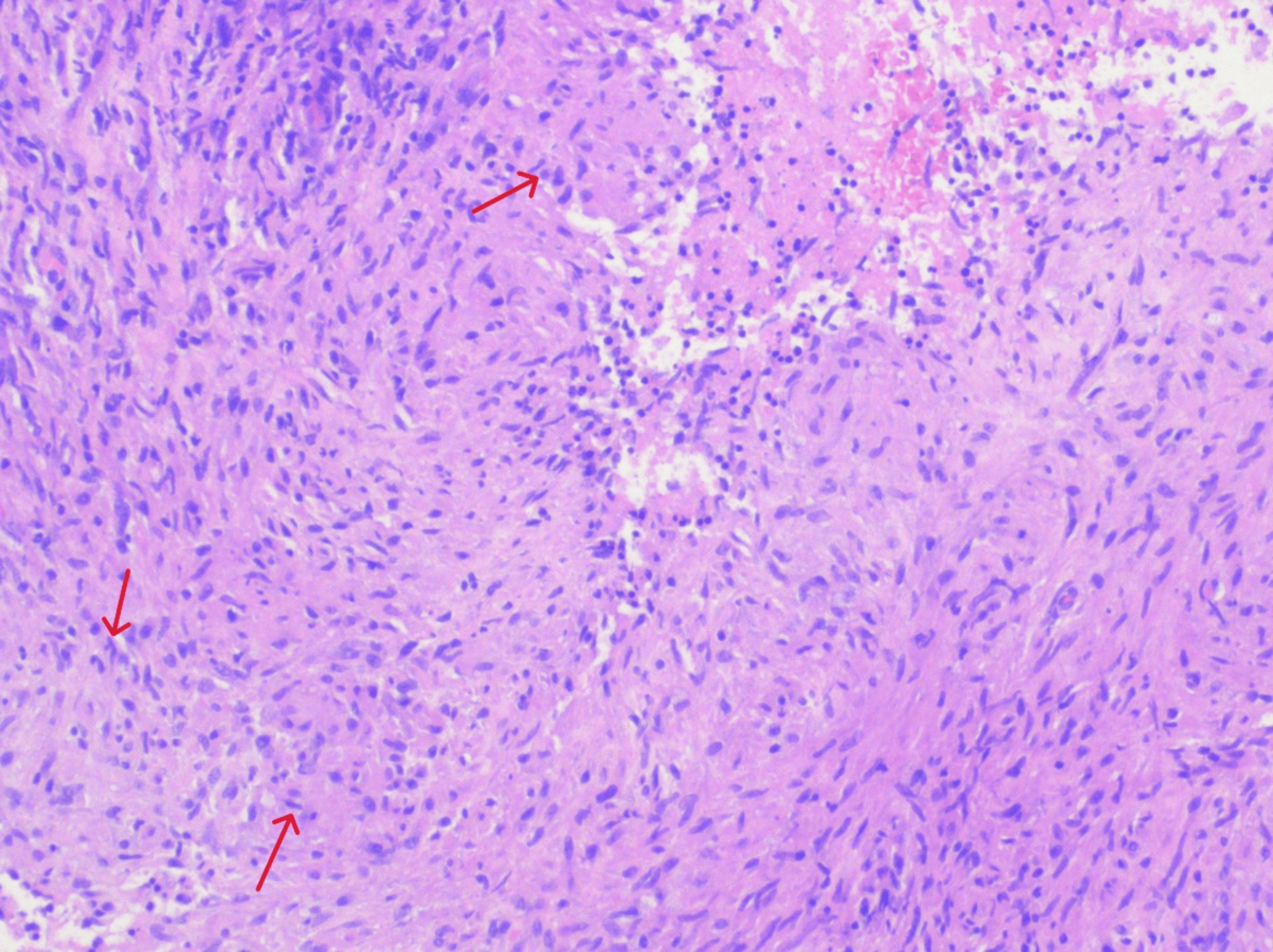
Right hepatic lobe PET-avid mass core needle biopsy showing liver parenchyma effaced by large, confluent, necrotizing granulomatous inflammation

**Figure 7:**
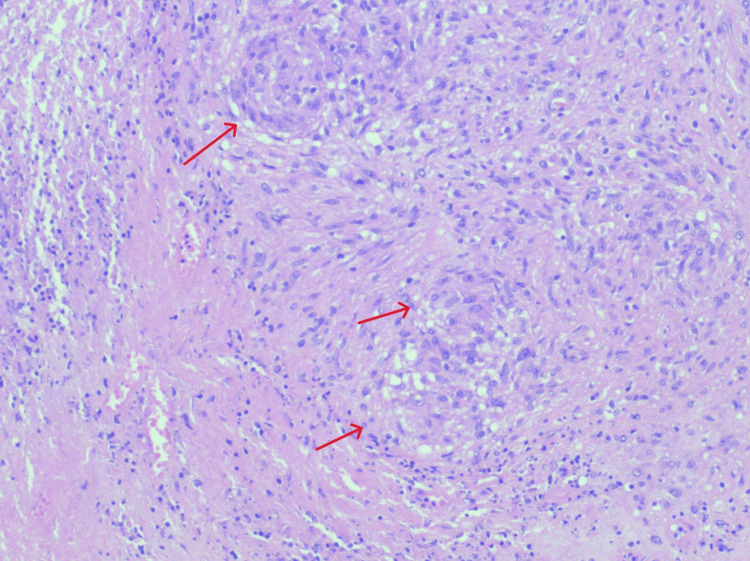
Right inguinal lymph node excision biopsy showing necrotizing granulomatous inflammation

Due to persistent symptoms, a repeat liver biopsy was performed, again demonstrating necrotizing granulomatous hepatitis (Figure 6). Right inguinal lymph node biopsy revealed similar findings (Figure 7). PCR testing on paraffin-embedded tissue (University of Washington) confirmed *B. henselae* DNA in both liver and lymph node samples. The patient confirmed cat ownership.

**Figure 8:**
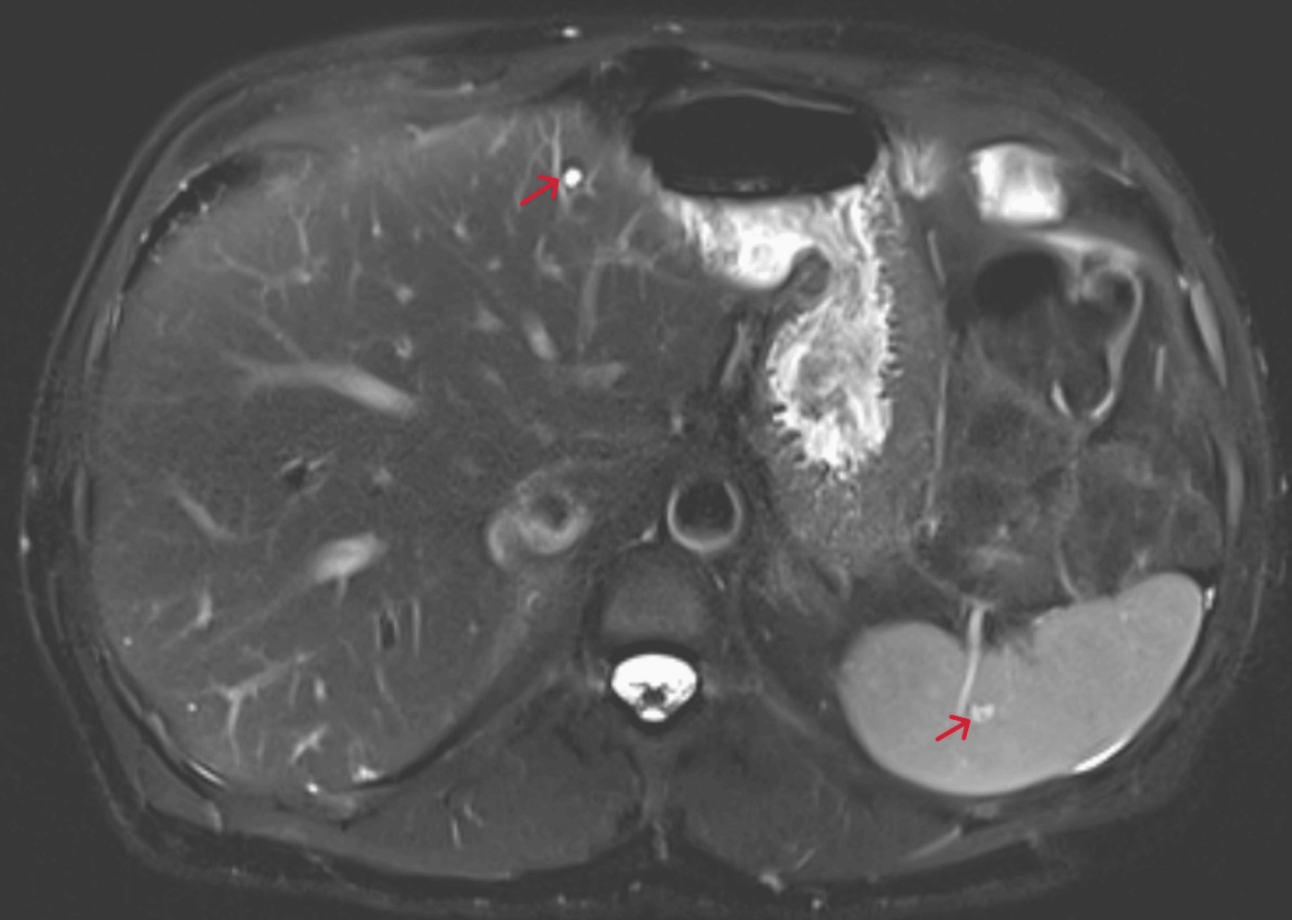
Follow-up abdominal MRI with and without contrast after antibiotic treatment, showing improved multifocal rim-enhancing lesions throughout the liver with a few residual scattered lesions. Splenic perfusion abnormality is noted

He was diagnosed with disseminated *B. henselae* infection and started on oral azithromycin 500 mg daily. After six weeks of antibiotic therapy, abdominal MRI showed improving hepatic lesions and new splenic findings, likely related to perfusion changes (Figure 8).

**Figure 9:**
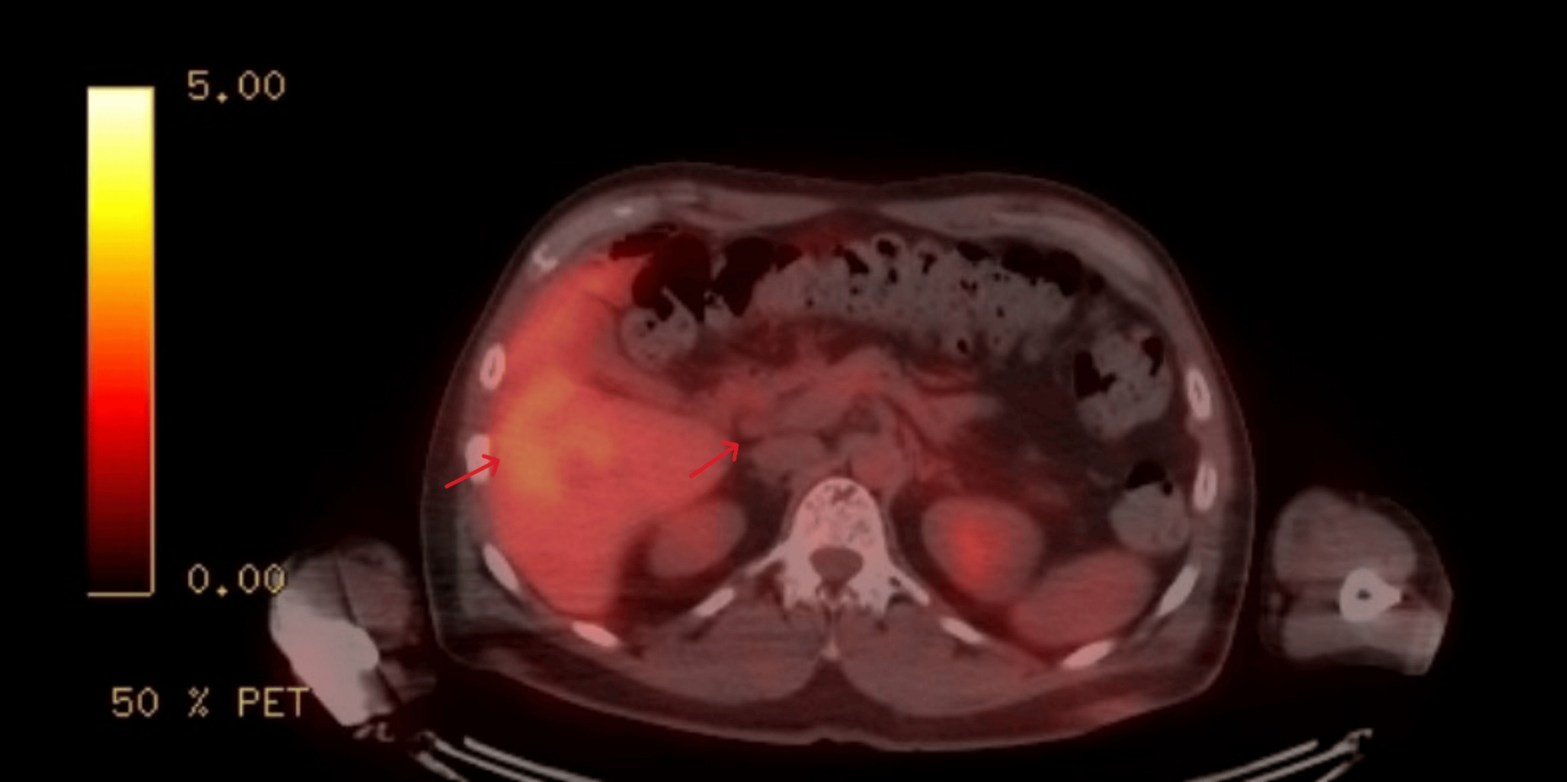
PET-CT after antibiotic therapy showing resolution of liver abnormalities and porta hepatis lymphadenopathy

**Figure 10:**
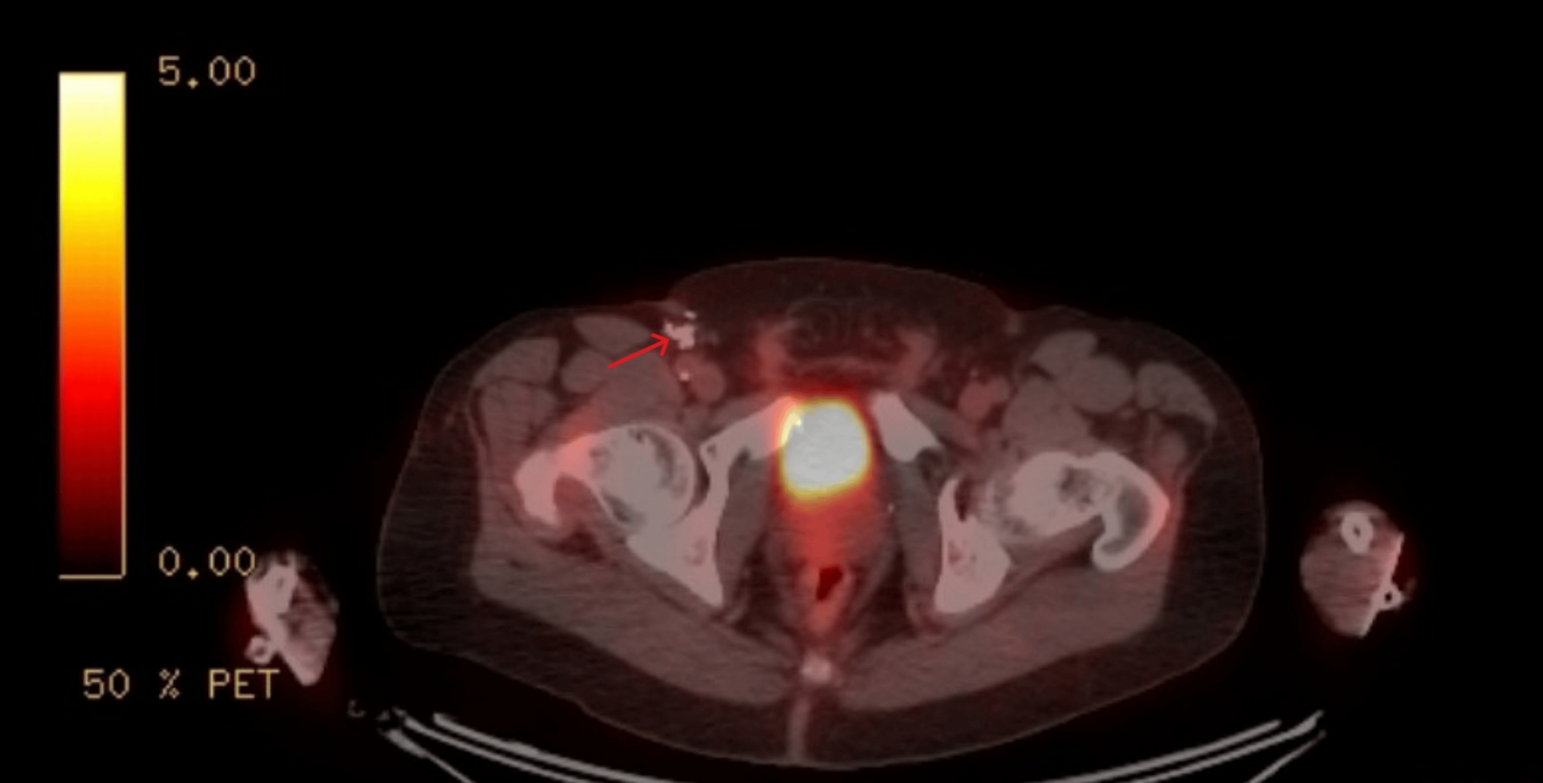
PET-CT after antibiotic therapy showing resolution of right inguinal lymphadenopathy, with post-surgical changes

Repeat PET-CT after completion of three months of antibiotic therapy showed resolution of FDG-avid lesions in the right hepatic lobe as well as FDG-avid portacaval and right inguinal lymph nodes, consistent with treatment of biopsy-proven granulomatous disease (Figure 9 and Figure 10). The patient reported complete symptomatic improvement, including resolution of fever, chills, and fatigue. His CRP returned to the normal range.

## Discussion

In this case, our patient with relapsed/refractory multiple myeloma presented after HD conditioning chemotherapy and ASCT with FUO, fatigue, and elevated inflammatory markers. His history was notable for exposure to a pet cat. Imaging revealed hepatosplenic involvement and lymphadenopathy due to systemic *Bartonella* infection. Biopsies of the liver and lymph nodes demonstrated necrotizing granulomas, and tissue PCR confirmed the diagnosis of systemic *Bartonella* infection. Prolonged antibiotic therapy resulted in symptomatic improvement and resolution of liver lesions and lymphadenopathy.

CSD typically presents as a self-limited, flu-like illness in immunocompetent individuals, characterized by fever, malaise, anorexia, fatigue, and headache [2]. A primary inoculation lesion at the scratch or bite site and painful regional lymphadenopathy usually appear 3-10 days and 1-3 weeks after inoculation, respectively [2].

In immunosuppressed transplant recipients, *Bartonella* may cause systemic infection involving visceral organs and deep lymph nodes [3,4]. These patients can develop bacteremia with prolonged fever and hematogenous dissemination, leading to complications such as osteomyelitis, culture-negative endocarditis, hemophagocytosis, neuroretinitis, and Parinaud oculo-glandular syndrome [3-6]. Angio-proliferative manifestations may include bacillary angiomatosis, peliosis hepatis, and splenic lesions [3-5,7].

Immunocompromised ASCT patients may have persistent false-negative or equivocal serologies due to impaired humoral immunity [7]. An IgG response has been documented even in transplant patients under maximal immunosuppression, and repeat serologies within 2-4 weeks to assess for a fourfold rise during convalescence are recommended, even if initial results are negative [7,8].

*Bartonella *is a fastidious organism, and tissue and blood cultures have low sensitivity [8]. Proactive communication with microbiology laboratory personnel is recommended so that culture techniques can be optimized and incubation periods extended to at least 21 days [7,8].

Positive Warthin-Starry staining, demonstrating clumps of extracellular gram-negative rods or L-shaped bacteria within areas of necrotic debris, can support the diagnosis of *Bartonella* infection [7]. Tissue biopsy with histopathology remains a key component in the diagnosis of bartonellosis [8].

*Bartonella *PCR on lymph node or abscess aspirates, or on biopsy specimens, provides the highest diagnostic sensitivity [7,8]. Necrotizing lymphadenitis and multiple caseating granulomas in visceral organs can be observed in immunosuppressed stem cell transplant patients [8].

CSD typically causes self-limited regional lymphadenopathy. In immunocompetent individuals with mild to moderate lymphadenitis, oral azithromycin shortens the duration of symptoms, reduces the risk of systemic disease, and prevents long-term sequelae [7-9].

Prolonged antibiotic therapy is often required in immunocompromised patients with disseminated disease [7,8]. The optimal duration of therapy is not well established in transplant patients and depends on the extent of immunosuppression, disease severity, and resolution of lesions [7,8]. In immunocompromised patients, macrolides, fluoroquinolones, doxycycline, aminoglycosides, and trimethoprim-sulfamethoxazole have been used for prolonged durations ranging from 4 to 32 weeks [6,10].

This case highlights the importance of maintaining a high index of suspicion for atypical infections such as *Bartonella *in immunosuppressed hematopoietic stem cell transplant patients presenting with FUO, particularly in those with known cat exposure. To our knowledge, this is the first reported case of disseminated *Bartonella* infection in a patient with relapsed/refractory multiple myeloma following ASCT.

## Conclusions

*B. henselae* infection should be considered in immunocompromised patients presenting with FUO, relapsing fever, granulomatous hepatitis, or persistent lymphadenopathy lasting more than three weeks, particularly in those with a history of chemotherapy, stem cell transplantation, or close contact with cats. Clinicians should routinely inquire about cat exposure when evaluating such cases. Standard serologic testing may be insufficient and can yield false-negative results in immunocompromised or post-ASCT patients. Early recognition and appropriate antimicrobial therapy are essential to reduce the risk of severe complications. Tissue PCR can facilitate rapid diagnosis in post-ASCT patients with persistent fever. Treatment of disseminated *Bartonella* infection in immunosuppressed individuals often requires prolonged antibiotic therapy, extending over several weeks to months. Combination antimicrobial regimens may be indicated in relapsed or refractory cases. Treatment duration and choice of agents should be individualized based on clinical response, with imaging studies employed to monitor therapeutic effectiveness.

## References

[1] Mira P, Theel ES (2024). Update on common Bartonella infections. Clin Microbiol Newsl.

[2] Florin TA, Zaoutis TE, Zaoutis LB (2008). Beyond cat scratch disease: widening spectrum of Bartonella henselae infection. Pediatrics.

[3] Bos F, Chauveau B, Ruel J (2022). Serious and atypical presentations of Bartonella henselae infection in kidney transplant recipients. Open Forum Infect Dis.

[4] Psarros G, Riddell J 4th, Gandhi T, Kauffman CA, Cinti SK (2012). Bartonella henselae infections in solid organ transplant recipients. Report of 5 cases and review of the literature. Medicine (Baltimore).

[5] Liston TE, Koehler JE (1996). Granulomatous hepatitis and necrotizing splenitis due to Bartonella henselae in a patient with cancer: case report and review of hepatosplenic manifestations of Bartonella infection. Clin Infect Dis.

[6] Lee RA, Ray M, Kasuga DT, Kumar V, Witherspoon CD, Baddley JW (2015). Ocular bartonellosis in transplant recipients: two case reports and review of the literature. Transpl Infect Dis.

[7] Morillas JA, Hassanein M, Syed B (2021). Early post-transplant cutaneous bacillary angiomatosis in a kidney recipient: case report and review of the literature. Transpl Infect Dis.

[8] Pischel L, Radcliffe C, Vilchez GA, Charifa A, Zhang XC, Grant M (2021). Bartonellosis in transplant recipients: a retrospective single center experience. World J Transplant.

[9] Bass JW, Freitas BC, Freitas AD (1998). Prospective randomized double blind placebo-controlled evaluation of azithromycin for treatment of cat-scratch disease. Pediatr Infect Dis J.

[10] Ahsan N, Holman M, Riley T, Abendroth C, Langhoff E. Yang H (1998). Peloisis hepatis due to Bartonella henselae in transplantation. A hemato-hepato-renal syndrome. Transplantation.

